# Draft Genome Sequence of Chromate-Resistant and Biofilm-Producing Strain *Pseudomonas alcaliphila* 34

**DOI:** 10.1128/genomeA.00125-12

**Published:** 2013-02-21

**Authors:** Luisa Santopolo, Emmanuela Marchi, Francesca Decorosi, Marco Galardini, Matteo Brilli, Luciana Giovannetti, Carlo Viti

**Affiliations:** a Dipartimento di Biotecnologie Agrarie, Sezione di Microbiologia, Università degli Studi di Firenze, Florence, Italy; b Dipartimento di Biologia Evoluzionistica, Università di Firenze, Florence, Italy; c UMR CNRS, 5558 Biométrie et Biologie Évolutive (LBBE), Lyon, France

## Abstract

We report the draft genome sequence of *Pseudomonas alcaliphila* 34, a Cr(VI)-hyperresistant and biofilm-producing bacterium that might be used for the bioremediation of chromate-polluted soils. The genome sequence might be helpful in exploring the mechanisms involved in chromium resistance and biofilm formation.

## GENOME ANNOUNCEMENT

*Pseudomonas alcaliphila* 34, previously identified as *Pseudomonas mendocina* with the Biolog-id system (Biolog, Hayward, CA) (1), is a Cr(VI)-hyperresistant and biofilm-producing bacterium isolated from a soil artificially polluted with chromate (2). The strain has been thoroughly characterized in terms of hundreds of biochemical attributes and its chromate-reducing capability in the presence of different carbon and energy sources (1), and in terms of its biofilm development capability and activity in the presence of several stressors (3). Therefore, *P. alcaliphila* 34 does indeed have exceptional abilities and it is a prospective candidate for remediation in environments where chromate contamination occurs together with other stressors (1).

Here, we present the draft genome sequence of *P. alcaliphila* 34 and provide information about the genetic bases that establish its high-level chromium tolerance and biofilm formation. Furthermore, its genome sequence might provide insight into the biotechnological exploitation of the organism for the remediation of chromium-contaminated sites (4).

The *P. alcaliphila* 34 genome was sequenced using Illumina HiSeq 2000 technology. A total of 101,323,720 reads (101 bp long) were assembled using the CLC Genomics Workbench assembler (CLC bio, Denmark), resulting in 1,718-fold coverage of a 5.44-Mb genome distributed in 277 unoriented contigs, and with an overall G+C content of 62%. BLAST (5) analysis on the nonredundant (NR) database allowed us to discard contaminant sequences from organisms unrelated to *Pseudomonas*, such as those from plants and insects.

Contigs were ordered using CONTIGuator 2.3 (6) with the *P. mendocina* NK01 genome, which is the closest available as a reference (GenBank accession no. CP002620.1). Fifty contigs, for a total of 5.27 Mb, were aligned with the reference genome, allowing us to define their relative order; sequences not mapped on the reference genome, corresponding to 13 contigs and a total of 144,392 nucleotides (nt), were added later on to the draft genome. Gaps between contigs were identified and closed using PCR followed by Sanger sequencing and, in some cases, primer walking sequencing of the PCR products. The final draft genome of *P. alcaliphila* 34 consists of eight supercontigs plus 10 single contigs, with a total length of 5,445,828 bp.

Genome annotation was performed with the Rapid Annotations using Subsystems Technology (RAST) pipeline (7), allowing for the identification of 4,983 protein-coding sequences, 61 tRNAs, and 4 copies of the genes for 5S, 16S, and 23S rRNA, as described for other *Pseudomonas* species (8, 9).

Genome analysis indicated that *P. alcaliphila* 34 possesses a putative *chrBACF* operon (10) that might be responsible for the high chromate resistance of the bacterium. Mercuric and arsenic resistance operons and many genes encoding putative multidrug resistance efflux systems were also identified on the genome. Additionally, genes known to be involved in biofilm formation, including the genes implicated in flagellar and type IV pilus biogenesis and motility (11, 12), plus two alginate biosynthesis gene clusters (13, 14), were identified.

A more detailed analysis of this genome and a comparative analysis with other Cr(VI)-resistant and biofilm-producing bacteria will provide further insight into the specific properties related to these processes.

### Nucleotide sequence accession numbers.

The draft genome sequence of *P. alcaliphila* 34 has been deposited at DDBJ/EMBL/GenBank under the accession no. ANGB00000000. The version described in this paper is the first version, ANGB01000000.1.
